# Tissue oxygen saturation is predictive of lactate clearance in patients with circulatory shock

**DOI:** 10.1186/s12871-023-02139-4

**Published:** 2023-05-25

**Authors:** Yan Chen, Jin-min Peng, Xiao-yun Hu, Shan Li, Xi-xi Wan, Rui-ting Liu, Chun-yao Wang, Wei Jiang, Run Dong, Long-xiang Su, Huai-wu He, Yun Long, Li Weng, Bin Du

**Affiliations:** 1 Medical Intensive Care Unit, State Key Laboratory of Complex Severe and Rare Diseases, Peking Union Medical College, Peking Union Medical College Hospital, Chinese Academy of Medical Sciences, Beijing, 100730 China; 2 Department of Critical Care Medicine, Peking Union Medical College, Peking Union Medical College Hospital, Chinese Academy of Medical Sciences, Beijing, 100730 China

**Keywords:** Tissue oxygen saturation, Shock, Lactate clearance, Diagnostic accuracy

## Abstract

**Background:**

Tissue oxygen saturation (StO_2_) decrease could appear earlier than lactate alteration. However, the correlation between StO_2_ and lactate clearance was unknown.

**Methods:**

This was a prospective observational study. All consecutive patients with circulatory shock and lactate over 3 mmol/L were included. Based on the rule of nines, a BSA (body surface area) weighted StO_2_ was calculated from four sites of StO_2_ (masseter, deltoid, thenar and knee). The formulation was as follows: masseter StO_2_ × 9% + (deltoid StO_2_ + thenar StO_2_) × (18% + 27%)/ 2 + knee StO_2_ × 46%. Vital signs, blood lactate, arterial and central venous blood gas were measured simultaneously within 48 h of ICU admission. The predictive value of BSA-weighted StO_2_ on 6-hour lactate clearance > 10% since StO_2_ initially monitored was assessed.

**Results:**

A total of 34 patients were included, of whom 19 (55.9%) had a lactate clearance higher than 10%. The mean SOFA score was lower in cLac ≥ 10% group compared with cLac < 10% group (11 ± 3 vs. 15 ± 4, p = 0.007). Other baseline characteristics were comparable between groups. Compared to non-clearance group, StO_2_ in deltoid, thenar and knee were significantly higher in clearance group. The area under the receiver operating curves (AUROC) of BSA-weighted StO_2_ for prediction of lactate clearance (0.92, 95% CI [Confidence Interval] 0.82-1.00) was significantly higher than StO_2_ of masseter (0.65, 95% CI 0.45–0.84; p < 0.01), deltoid (0.77, 95% CI 0.60–0.94; p = 0.04), thenar (0.72, 95% CI 0.55–0.90; p = 0.01), and similar to knee (0.87, 0.73-1.00; p = 0.40), mean StO_2_ (0.85, 0.73–0.98; p = 0.09). Additionally, BSA-weighted StO_2_ model had continuous net reclassification improvement (NRI) over the knee StO_2_ and mean StO_2_ model (continuous NRI 48.1% and 90.2%, respectively). The AUROC of BSA-weighted StO_2_ was 0.91(95% CI 0.75-1.0) adjusted by mean arterial pressure and norepinephrine dose.

**Conclusions:**

Our results suggested that BSA-weighted StO_2_ was a strong predictor of 6-hour lactate clearance in patients with shock.

**Supplementary Information:**

The online version contains supplementary material available at 10.1186/s12871-023-02139-4.

## Introduction

Circulatory shock is a life-threatening condition affecting about one-third of patients admitted to intensive care unit (ICU) [1]. In such patients, hyperlactatemia has been considered as a signal of tissue hypoperfusion and associated with poor outcome [2, 3]. Meanwhile, the decrease in lactate level is believed to be associated with improved outcome in shock, including septic shock [4] and cardiogenic shock [5]. However, lactate-guided resuscitation might lead to fluid overload with increased risk of morbidity and mortality because of delayed lactate decrease in patients with normalized tissue perfusion [6].

Near-infrared spectroscopy is a technique to determine tissue oxygen saturation (StO_2_) by identifying oxygenated and deoxygenated hemoglobin with different light abortion patterns. Similarly, a decrease of StO_2_ level is a reliable indicator for tissue hypoperfusion in trauma patients [7–9]. Furthermore, a recent study showed that StO_2_ alterations could appear earlier than lactate alteration in a sheep model of peritonitis [10]. The muscle StO_2_ significantly decreased soon after 8 h from sepsis induction, which was 20 h earlier than the elevation of lactate.

Thus, StO_2_ might have predictive value on lactate decrease, but the clinical implications remained uncertain. Previous prospective observational studies demonstrated the correlation between lactate and StO_2_ from different anatomical sites including knee [11], cerebral [12] and thenar StO_2_ [13]. Ait-Oufella et al. observed that knee StO_2_ was associated with lactate level (R^2^ = 0.2, P < 0.002) after 6 h of septic shock resuscitation [11]. Tayar et al. showed significant correlation between cerebral regional oxygen saturation and lactate in shock at 8, 24, 48, and 72 h from admission [12].

One previous study aimed to evaluate the predictive value of thenar StO_2_ for lactate clearance in patients after cardiac surgery without focusing on hypotension [14]. Overall, all the studies failed to show the correlation between StO_2_ and lactate clearance in shock. This could be partly attributable to redistributes flow preferentially to vital organs during shock [15]. Accordingly, sublingual microcirculation fails to predict gut mucosal microcirculation in septic patients. This means that microcirculation (StO_2_ included) status at one location can be only used to indicate local perfusion alterations instead of global perfusion [16].

So far, there is no specific method to provide a general evaluation of microcirculation. The most common site of StO_2_ was thenar [17]. However, it has been suggested that StO_2_ may have better predictive value in site of masseter [18], deltoid [18], and knee [11]. The rules of nines is known as a tool used to assess the total body surface area involved in burn patients. And it has also been used to evaluate the area of muscle injury for assessment of severity of traumatic rhabdomyolysis in patients with Crush syndrome [19]. Similarly, general StO_2_ could be estimated according to the rule of nines. Masseter StO_2_ could represent head, accounting for 9%, deltoid and thenar StO_2_ represent arms and torso, accounting for 45%, and knee StO_2_ represent legs, accounting for 46%. We thus hypothesized that BSA (body surface area) -weighted StO_2_, which was generated from four different sites of StO_2_ was associated with lactate decrease in patients with shock.

## Method

We conducted a prospective observational study in a 15-bed medical ICU in a tertiary teaching hospital. The study protocol was approved by the institutional review board of Peking Union Medical College Hospital. Informed consents were obtained from the patients or relatives.

### Study Population

All consecutive patients admitted for circulatory shock with a serum lactate level of 3.0 mmol/L or more were included. Circulatory shock was defined as systolic blood pressure less than 90 mm Hg or mean arterial pressure was less than 70 mm Hg, patients with evidence of tissue hypoperfusion (including but not limited to oliguria, skin mottling, altered mental status, cool peripheries, hyperlactatemia, etc.) [20]. All patients younger than 18 years old or pregnancy were excluded.

### Investigated parameters

Demographic data, chronic comorbidities, Sequential Organ Failure Assessment (SOFA), shock type, and infection site were recorded on admission. Four sites of StO_2_ (masseter, deltoid, thenar, and knee), vital signs, blood lactate (arterial), arterial and central venous blood gas were recorded simultaneously within 48 h of ICU admission. And patients were still in state of shock at the moment of measurement after resuscitation was complete according to the Surviving Sepsis Guidelines [21]. Blood lactate concentration was measured repeatedly after 6 h from baseline when StO_2_ was initially monitored. A central line was placed in internal jugular vein in patients to allow for central venous blood sampling. Radial artery or femoral artery was cannulated in all patients for invasive blood pressure monitoring (IntelliVue Patient Monitor MP 70 (Philips Medical System, Boeblingen, Germany). Arterial and venous blood gases with lactate were measured immediately using GEM Premier 4000 blood gas analyzer (Instrumentation Laboratory, Bedford, Mass). StO_2_ was measured at right side of the masseter, deltoid, thenar, and knee sites by the Noninvasive cerebral oximetry monitor, BRS-1 with four 40-mm depth infrared probes (Casibrain Techonology Inc, Beijing, CHN). The StO_2_ values were recorded after 1 min of measurement when the signal was stable. Survival was followed-up during 14 days.

### Definitions

Clearance of Lactate (cLac) was calculated as a change in blood lactate levels (%) after 6 h from baseline when StO2 was initially monitored [22]. The formula is as follows:

[(0 h-Lactate − 6 h-Lactate)/ 0 h-Lactate] × 100%. A positive value indicates a decrease in lactate rate.

Additionally, patients were divided into lactate clearance group and lactate non-clearance group. Lactate clearance was defined as 6-hour lactate clearance more than 10% [22].

Mean StO_2_ was the mean value of the four sites StO_2_. A BSA-weighted StO_2_ was calculated from four sites of StO_2_ (masseter, deltoid, thenar and knee), based on the rules of nines, which is a method used to quantify the area of affected skin in burns victims [23]. Masseter StO_2_ represented head, accounting for 9%, deltoid and thenar StO_2_ represented arms and torso, accounting for 45%, and knee StO_2_ represented legs, accounting for 46% (Fig S1).

The formulation was as follows:

masseter StO_2_ × 9% + (deltoid StO_2_ + thenar StO_2_) × (18% + 27%)/ 2 + knee StO_2_ × 46%

The Septic Shock 3.0 definition was used to define septic shock in the study [24].

### Statistical analysis

On the basis of previous study, area under the receiver operating curves (AUROC) of StO_2_ for prediction of lactate clearance was expected to be 0.8^14^. Total sample size required was 34 (17 in each group), with a power of 90% and α = 5% (two-sided). Values were presented as the mean (SD) or median (interquartile range (IQR)) for continuous variables as appropriate and as percent for categorical variables. Comparisons between groups were made using the chi-square test or Fisher’s exact test for categorical variables and Student’s t-test or the Mann–Whitney U test for continuous variables, as appropriate. All correlations among parameters were calculated as Spearman’s correlation, including correlation between StO_2_ in different sites, as well as correlations between StO_2_ , lactate clearance, MAP and norepinephrine dose. We evaluated correlations of StO2 in different sites using Spearman rank coefficients and visualized the relationships with heatmap. AUROC curves for lactate clearance was computed using the trapezoidal rule. The confidence interval (CI) were determined by the bootstrap technique, and comparison was made as described in DeLong [25]. The analysis of ROC is corrected for confounding factors including norepinephrine dose and mean arterial pressure (MAP). The category-free net reclassification improvement (NRI) was performed to quantify improvement offered by BSA-weighted StO_2_ [26]. Subgroup analysis was conducted based on patients with septic shock. All statistical analyses were performed using R (version 4.0.0, R studio, Boston, MA). GraphPad Prism 9.0 was used to graph results.

## Result

### Study population

From April 2021 to April 2022, 34 patients were included, of whom 19 (55.9%) had a lactate clearance ≥ 10%. The baseline characteristics of the two groups were shown in Table 1. The most common type of shock was septic shock, followed by cardiogenic shock, and hypovolemic shock. The two main sites of infection were lung (26%) and bloodstream (12%). All of the patients were treated with norepinephrine, median dose 0.5 (interquartile 0.3–1.0) ug/kg/min. Four (12%) patients were treated with epinephrine, median dose 0.3 (interquartile 0.2–0.3) ug/kg/min. The mean SOFA score was lower in cLac ≥ 10% group compared with cLac < 10% group (11 ± 3 vs. 15 ± 4, p = 0.007). Other baseline characteristics were comparable between groups. The 14-day mortality was lower in cLac ≥ 10% group (21% vs. 60%, p = 0.049).

**Table 1:** Characteristics of patients

	All patients (n = 34)	cLac ≥ 10% (n = 19)	cLac < 10% (n = 15)	*p* value ^*^
Age, years	51 ± 17	51 ± 18	50 ± 17	0.850
Male, n (%)	16 (47)	10 (53)	6 (40)	0.699
BSA, m^2^	1.8 ± 0.2	1.9 ± 0.2	1.7 ± 0.2	0.003
SOFA score	13 ± 4	11 ± 3	15 ± 4	0.007
Comorbidities, n (%)	25 (74)	13 (68)	12 (80)	0.713
Hypertension	7 (21)	3 (16)	4 (27)	-
Coronary artery disease	4 (12)	3 (16)	1 (7)	-
Chronic pulmonary disease	2 (6)	1 (5)	1 (7)	-
Malignancy	6 (18)	4 (21)	2 (13)	-
Diabetes mellitus	7 (21)	5 (26)	2 (13)	-
Chronic kidney disease	4 (12)	2 (11)	2 (13)	-
Others§	6 (18)	2 (11)	4 (27)	-
Type of shock, n (%)				0.486
Septic shock	24 (71)	12 (63)	12(80)	-
Cardiogenic shock	7 (21)	4 (21)	3 (20)	-
Hypovolemic shock	4 (12)	4 (21)	0 (0)	-
Unknown	3 (9)	2 (11)	1 (7)	-
Infection site, n (%)				0.155
Lung	9 (26)	6 (32)	3 (20)	-
Abdomen	3 (9)	1 (5)	2 (13)	-
Urinary tract	3 (9)	2 (11)	1 (7)	-
Soft tissue	2 (6)	2 (11)	0 (0)	-
Bloodstream	4 (12)	2 (11)	2 (13)	-
Others^Δ^	4 (12)	0 (0)	4 (27)	-
Mechanical ventilation, n (%)	24 (71)	12 (63)	12 (80)	0.489
Renal replacement therapy, n (%)	8 (24)	3 (16)	5 (33)	0.429
14-day mortality, n (%)	13 (38)	4 (21)	9 (60)	0.049
ICU length of stay, days^¶^	4.0 (1.2–10.5)	6.0 (2.5–9.5)	2.0 (1.0–12.0)	0.151

*cLac* Lactate clearance; *BSA* body surface area; *SOFA* Sequential Organ Failure Assessment

¶ Data presented as median (interquartile)

§ Other comorbidities include rheumatic and hematological diseases

^Δ^ Other infection site include central nervous system, intrathoracic and biliary tract infection

^*^ Comparisons were made using ANOVA test for continuous variables and Chi-Squared test for categorical varia

### Hemodynamic parameters assessment

Hemodynamic parameters assessments were showed in Table 2. The 0-hour lactate concentration in cLac ≥ 10% group was 4.9 ± 2.0 mmol/L and 7.7 ± 4.6 mmol/L in cLac < 10% group, with a lactate clearance 39.1 ± 17.4% and − 32.3 ± 38.1%, respectively.

**Table 2:** Hemodynamics characteristics of patients

	All patients (n = 34)	cLac ≥ 10% (n = 19)	cLac < 10% (n = 15)	p value *
Heart rate, bpm	114 ± 22	110 ± 20	119 ± 25	0.230
MAP, mmHg	81 ± 13	79 ± 14	84 ± 12	0.308
Vasopressor				
Norepinephrine, n (%)	34 (100)	19 (100)	15 (100)	1.000
Norepinephrine, µg/kg/min^¶^	0.5 (0.3–1.0)	0.5 (0.3–0.9)	0.8 (0.3–1.3)	0.555
Epinephrine, n (%)	4 (12)	1 (5)	3 (20)	0.431
Epinephrine, µg/kg/min^¶^	0.3 (0.2–0.3)	0.3 (0.3–0.3)	0.3 (0.2–0.3)	1.000
Urinary output, ml/h	62 ± 73	86 ± 86	32 ± 36	0.030
Lactate initial, mmol/L	6.2 ± 3.6	4.9 ± 2.0	7.7 ± 4.6	0.022
6 h- Lactate, mmol/L	6.0 ± 5.2	3.0 ± 1.3	9.9 ± 5.7	< 0.001
cLac, %	7.6 ± 45.6	39.1 ± 17.4	-32.3 ± 38.1	< 0.001
StO_2_, %				
masseter	73.6 ± 4.3	74.6 ± 4.0	72.4 ± 4.4	0.132
deltoid	74.9 ± 5.5	76.9 ± 4.1	72.2 ± 6.1	0.011
thenar	68.3 ± 6.6	70.8 ± 3.7	65.2 ± 8.1	0.012
knee	69.5 ± 7.2	73.1 ± 4.4	64.7 ± 7.5	< 0.001
weighted	71.0 ± 4.9	73.6 ± 2.8	67.5 ± 5.1	< 0.001
mean	71.8 ± 4.2	73.9 ± 2.6	69.1 ± 4.4	< 0.001
ScvO_2_, %	64.4 ± 12.9	66.9 ± 9.4	60.3 ± 17.2	0.264

*cLac* Lactate clearance;*MAP* mean arterial pressure, *StO2* tissue oxygen saturation, *ScvO*_*2*_ central venous oxygen saturation

¶ Data presented as median (interquartile)

^*^ Comparisons were made using ANOVA test for continuous variables and Chi-Squared test for categorical variables

For StO_2_ measurement, there was one aberrant value at knee site (not detectable) and was excluded from analyses. Overall, the StO_2_ value vary considerably in different anatomical sites. Deltoid and masseter StO_2_ were higher than knee and thenar (deltoid 74.9 ± 5.5; masseter 73.6 ± 4.3; knee 69.5 ± 7.2; thenar 68.3 ± 6.6). As for comparisons of StO_2_ between different types of shock, there is a tendency existed toward lower StO_2_ of thenar, knee and weighted in cardiogenic shock than other types of shock (Table S1). No difference was seen in StO_2_ sites of masseter and deltoid. Compared to cLac < 10% group, all sites of StO_2_ except masseter were significantly higher in cLac ≥ 10% group. BSA-weighted of the four sites StO_2_ was also higher in the cLac ≥ 10% group than cLac ≥ 10% group (73.6 ± 2.8 vs. 67.5 ± 5.1, p < 0.001).

Mean arterial pressure, heart rate, vasopressor doses and ScvO_2_ did not differ between two groups. Fluid balances were lower in cLac ≥ 10% group than in cLac < 10% group 2 and 6 h after StO_2_ measurement (p = 0.042; p = 0.031) (Fig S2).

### Correlations between StO_2_ and hemodynamic parameters

No significant correlation exists between five sites of StO_2_(Fig S3). All sites of StO_2_ were negatively correlated with MAP, while no correlation was found between StO_2_ and norepinephrine dose (Table S2).

**Fig. 1:**
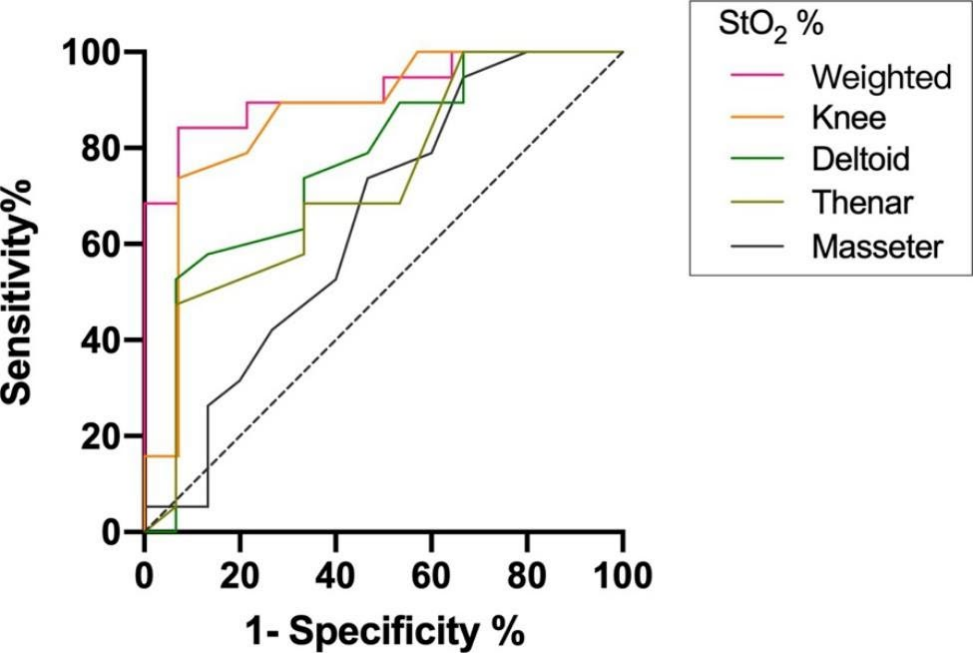
ROC curves. Weighted, masseter, deltoid, thenar, knee StO2 according to 6-hour lactate clearance. The AUROCs are 0.92 (0.82–1.00), 0.65 (0.45–0.84), 0.77 (0.60–0.94),0.72 (0.55–0.90) and 0.87 (0.73–1.00), respectively

There were significant correlations between lactate clearance and knee, deltoid and BSA-weighted StO_2_ (Table S3). Hemodynamic indicators include central venous oxygen saturation (ScvO_2_), mean arterial pressure and masseter StO_2_ were not predictive of lactate clearance (area under the ROC curve was < 0.7). The area under the receiver operating curves (AUROC) of BSA-weighted StO_2_ for prediction of lactate clearance (0.92, 95% CI [Confidence Interval] 0.82-1.00) was significantly higher than StO_2_ of masseter (0.65, 95% CI 0.45–0.84; p < 0.01), deltoid (0.77, 95% CI 0.60–0.94; p = 0.04), thenar (0.72, 95% CI 0.55–0.90; p = 0.01), and similar to knee (0.87, 0.73-1.00; p = 0.40), mean StO_2_ (0.85, 0.73–0.98; p = 0.09) (Fig. 1; Table 3). Choosing a threshold of BSA-weighted StO_2_ of at least 72% was associated with a sensitivity of 84% and a specificity of 93% to predict lactate clearance. The predictive positive value was 89% (over 72%, 16/18 patients showed lactate clearance more than 10%), to be compared with 20% (3/15) in patients with BSA-weighted StO_2_ of lower than 72%.

**Table 3:** Area under the ROC curves for predicting 6-hour lactate clearance

Patients		AUROC (95% CI)	threshold	sensitivity	specificity
StO_2_, %					
masseter	all patients (n = 34)	0.65 (0.45–0.84)	71	0.95	0.33
	septic shock (n = 24)	0.61 (0.37–0.85)	71	0.92	0.33
deltoid	all patients (n = 34)	0.77 (0.60–0.94)	78	0.53	0.93
	septic shock (n = 24)	0.79 (0.57–0.99)	77	0.75	0.83
thenar	all patients (n = 34)	0.72 (0.55–0.90)	73	0.47	0.93
	septic shock (n = 24)	0.69 (0.48–0.91)	70	0.75	0.58
knee	all patients (n = 33)	0.87 (0.73–1.00)	71	0.74	0.93
	septic shock (n = 23)	0.79 (0.60–0.98)	71	0.58	0.91
weighted	all patients (n = 33)	0.92 (0.82–1.00)	72	0.84	0.93
	septic shock (n = 23)	0.84 (0.67–1.00)	72	0.75	0.91
mean	all patients (n = 33)	0.85 (0.73–0.98)	72	0.89	0.71
	septic shock (n = 23)	0.81 (0.62–0.99)	74	0.58	1.00
ScvO_2_, %	all patients (n = 21)	0.58 (0.27–0.89)	53	0.92	0.50
	septic shock (n = 15)	0.53 (0.17–0.88)	72	0.78	0.50
Urine output, ml/h	all patients (n = 34)	0.71 (0.54–0.89)	53	0.63	0.73
	septic shock (n = 24)	0.70 (0.49–0.92)	80	0.5	0.92
MAP, mmHg	all patients (n = 34)	0.65 (0.45–0.84)	80	0.63	0.8
	septic shock (n = 24)	0.59 (0.34–0.84)	80	0.58	0.75

*AUROC (95%CI)* area under the receiver operating curves (95% Confidence Interval), *PPV* positive predictive value, *NPV* negative predictive value, *StO2* tissue oxygen saturation, *ScvO*_*2*_ central venous oxygen saturation, *MAP* mean arterial pressure

**Fig. 2:**
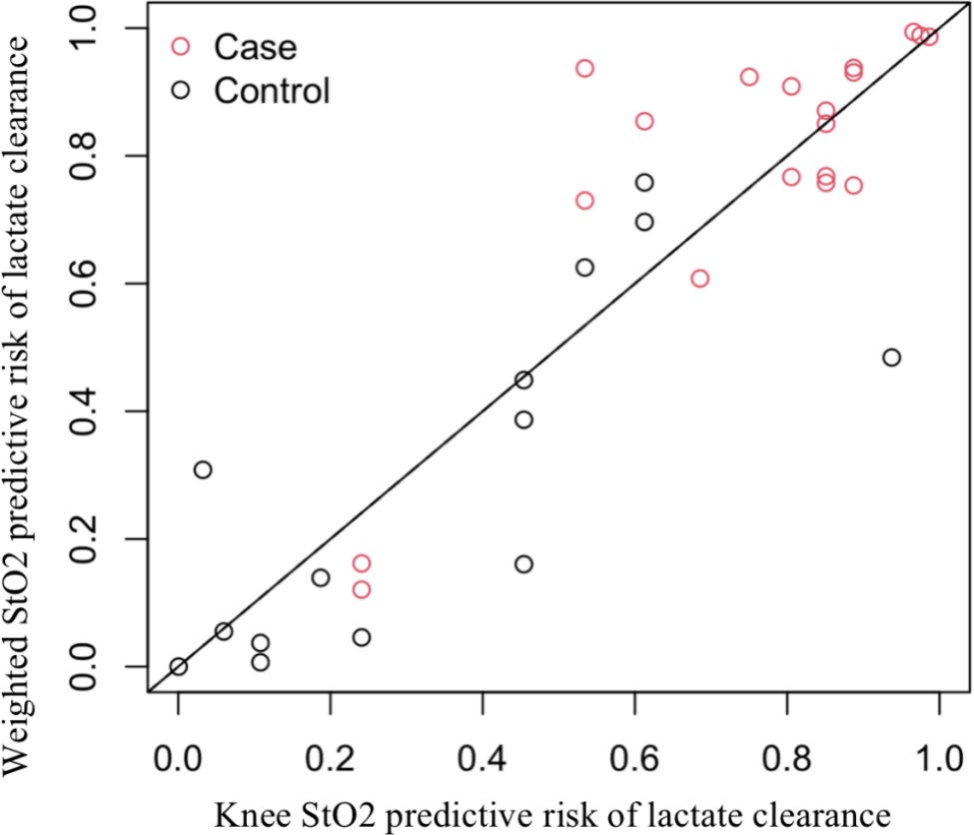
Predicted probabilities by knee StO2 and weighted StO2 with diagonal line showing the comparable predicted probabilities in lactate clearance group and non-clearance group The red circles represent the lactate clearance group (case) and the white circles represent the non-clearance group (control). Circles above the diagonal line indicate an increase in the probability of correct prediction of weighted StO2 compared to knee StO2.

As shown in Fig. 2 and Fig S4, BSA-weighted StO_2_ had probabilities reclassified upwards over the knee StO_2_ and mean StO_2_ model for cLac ≥ 10% group (52.6% and 73.7%, respectively) and for cLac < 10% group (28.6% and 28.6%, respectively). Overall, BSA-weighted StO_2_ model had continuous net reclassification improvement over the knee StO_2_ and mean StO_2_ model (48.1% and 90.2%, respectively). The AUROC for BSA-weighted StO_2_ was 0.91(95%CI 0.75 -1.0) adjusted by mean arterial pressure and norepinephrine dose (Fig S5).

A total of 24 patients were included in the septic shock subgroup. The BSA-weighted StO_2_ have the largest areas under the curves [0.84, 95%CI (0.67–1.00)] for predicting 6-hour lactate clearance in the septic shock subgroup (Table 3).

## Discussion

In this prospective observational study, BSA-weighted and knee StO_2_ are predictive of lactate clearance in patients with shock. In addition, BSA-weighted StO_2_ demonstrated better accuracy to predict lactate clearance than knee StO_2_. The result remained robust after adjusted by mean arterial pressure and norepinephrine dose and in subgroup analysis of patients with septic shock. BSA-weighted StO_2_ over 72% indicated a subsequent normalization of lactate within 6 h.

StO_2_ values varied in different sites, with deltoid and masseter higher than knee and thenar. On the other hand, no significant correlation was found between all sites of StO_2_. This might be attributable to the maldistribution of the blood flow to maintain normal blood flow to the vital organ during shock [27–29]. In a prospective observational study with 22 septic shock patients included, no correlation between basal intestinal or sublingual microcirculation and response to a fluid challenge was found [15]. The study suggested a dissociation between sublingual and intestinal microcirculation during shock. Since there are dissociations between microcirculation, assessment of microcirculatory at certain site can only represent the local microcirculation. Accordingly, the tissue oxygen saturation in any single site might not be considered as an indicator of whole-body perfusion. Two studies suggested forearm StO_2_ is a more sensitive parameter to hypovolemia than thenar StO_2_ [30, 31]. Additionally, a systematic review of StO_2_ monitoring in shock suggested better mortality prediction in sites of knee and brachial muscle, compared to thenar muscle [17]. From this perspective, single site monitoring of microcirculation may limit the predictive value of indicators like StO_2_.

However, most of the studies conducted with single site monitoring of StO_2_ due to the limited number of probes [17]. Ait-Oufella et al. used simultaneous measurements from thenar and knee only for comparison of two sites of StO_2_ [11]. Colin et al. monitored masseter, deltoid and thenar StO_2_ at the same time and mean value of the them was proposed as a surrogate of ScvO_2_^18^. Authors reported correlations between ScvO_2_ and masseter, deltoid, thenar StO_2_, and mean value of StO_2_ in three sites during 6-hour early resuscitation in patients with severe sepsis. However, knee StO_2_, which was considered as a good predictor of tissue perfusion, was missed [11]. Furthermore, simple average applied in previous study is lack of sufficient microcirculation representative since StO_2_ at different sites had considerable heterogeneity. Instead, in our study, the calculated BSA-weighted StO_2_ had taken weight of four important parts of systemic microcirculation into consideration.

Experimental studies found skeletal muscle PO_2_ monitoring at quadriceps femoris muscle using a polarographic needle electrode was sensitive to the hemodynamic changes during various types of shock [28]. Skeletal muscle PO_2_ reduced rapidly early before hypotension occurred. This suggested that microcirculation dysfunction could appear earlier than macrocirculation, which gives ground to the consideration of predictive value of StO_2_ for lactate decrease. Previous observational studies have suggested that dynamic StO_2_ alteration may be associated with lactate clearance in shock. Lima et al. reported patients with persistent lower thenar StO_2_ (< 70%) had lower lactate clearance in early resuscitation of septic shock [13]. Ait-Oufella et al. also observed the change of knee StO_2_ between 6 and 24 h after septic shock initiation was associated with lactate clearance [11]. Besides, the predictive value of StO_2_ for lactate clearance have been discussed in patients after surgery. Kopp et al. found minimum thenar StO_2_ is a predictor of lactate clearance in post cardiac surgery patients with an AUROC of 0.83 [14]. This was consistent with our findings that deltoid, thenar, knee and BSA-weighed StO_2_ were predictive of lactate decrease in patients with circulatory shock. In addition, BSA-weighted StO_2_ showed greatest AUC than any other single site of StO_2_. In clinical situation, when patient is still in a state of shock after resuscitation, the changing trends of lactate are unknown at the moment. If BSA-weighted StO_2_ value is over 72% at the moment, then his lactate level is more likely to decrease over 10% within 6 h. This would be helpful for guiding for following treatment.

Different doses of norepinephrine and mean arterial pressure (MAP) might have effects on StO_2_ despite a considerable interindividual variation [32, 33]. In our study, MAP and vasopressor dose did not differ between two groups. Also, the BSA-weighted StO_2_ diagnostic performance for lactate clearance was unchanged after controlling for norepinephrine doses and MAP. Fluid was another treatment which might affect StO_2_ value. However, the lower fluid balance in the lactate clearance group have ruled out the possibility.

Our study has strengths. The simultaneously monitoring of microcirculation among multiple sites was performed in the study. Accordingly, BSA-weighted StO_2_ calculated on four sites StO_2_ was generated. Unlike single sites StO_2_ measured in previous studies, BSA-weighted StO_2_ was a potential indicator of macrocirculation.

Our study has limitations. Firstly, this is a single-center study from a tertiary hospital with a relatively small sample size. Secondly, the heterogeneity of patients enrolled may indicate selection bias. However, heterogenous microcirculatory alterations were documented in sepsis [34] as well as in traumatic hemorrhagic shock [35], and cardiogenic shock [36]. Previous study has shown the predictive value of near-infrared spectroscopy derived StO_2_ for various types of shock [17]. Moreover, subgroup analysis of septic shock patients in our study remained robust. Further investigation for specified population was warranted, though. Thirdly, exclusion of lactate concentration lower than 3mmol/L limits the generalizability of our findings. However, patients in this subgroup might benefit less from serial lactate monitoring [37].

## Conclusion

Our results suggest that StO_2_ was a predictor of lactate clearance in patients with shock. The blood lactate concentrations of patients with a BSA-weighted StO_2_ over 72% are more likely to decrease in the next 6 h.

## Electronic supplementary material

Below is the link to the electronic supplementary material.

Supplementary Material 1

## Data Availability

The datasets analyzed during the current study are available from the corresponding author on reasonable request.
