# Supplementary material for: Tissue oxygen saturation is predictive of lactate clearance in patients with circulatory shock
Source: BMC Anesthesiol. 2023 May 25;23:179. doi: 10.1186/s12871-023-02139-4 (PMC10210288; doi:10.1186/s12871-023-02139-4)
Supplement: Supplementary file 1 — Supplementary Material 1 [file 12871_2023_2139_MOESM1_ESM.docx]

Supplementary appendix to

Tissue oxygen saturation is predictive of lactate clearance in patients with circulatory shock

TABLE OF CONTENTS OF SUPPLEMENTAL ELECTRONIC MATERIAL

[Figures 3](#_Toc134414198)

[Fig S1. Schematic diagram of four sites of tissue oxygen saturation and microcirculatory sites they represent. 3](#_Toc134414199)

[Fig S2. Fluid balance after measurement of tissue oxygen saturation 4](#_Toc134414200)

[Fig S3. Correlations between tissue oxygen saturation 5](#_Toc134414201)

[Fig S4. Predicted probabilities by mean StO_2_ and BSA-weighted StO_2_ with diagonal line showing the comparable predicted probabilities in lactate clearance group and non-clearance group. 6](#_Toc134414202)

[Fig S5. Adjusted ROC curve. 7](#_Toc134414203)

[Tables 8](#_Toc134414204)

[Table S1 Comparisons of tissue oxygen saturation between different types of shock 8](#_Toc134414205)

[Table S2 Correlations between tissue oxygen saturation and MAP, norepinephrine dose 8](#_Toc134414206)

[Table S3 Correlations between tissue oxygen saturation and lactate clearance 8](#_Toc134414207)

# Figures


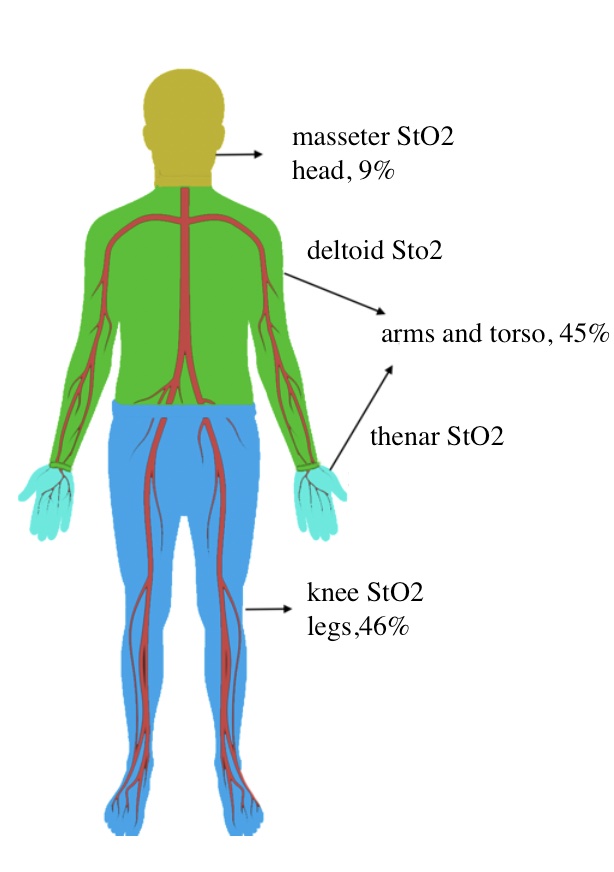


## Fig S1. Schematic diagram of four sites of tissue oxygen saturation and microcirculatory sites they represent.


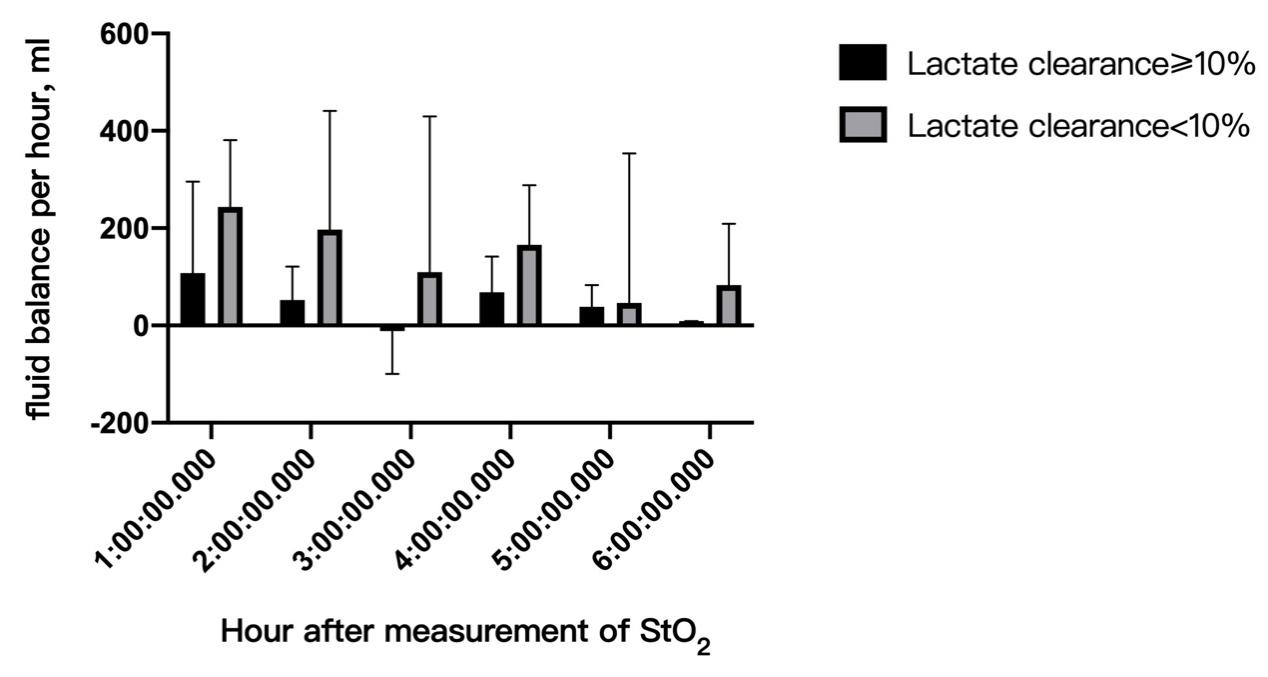


## Fig S2. Fluid balance after measurement of tissue oxygen saturation


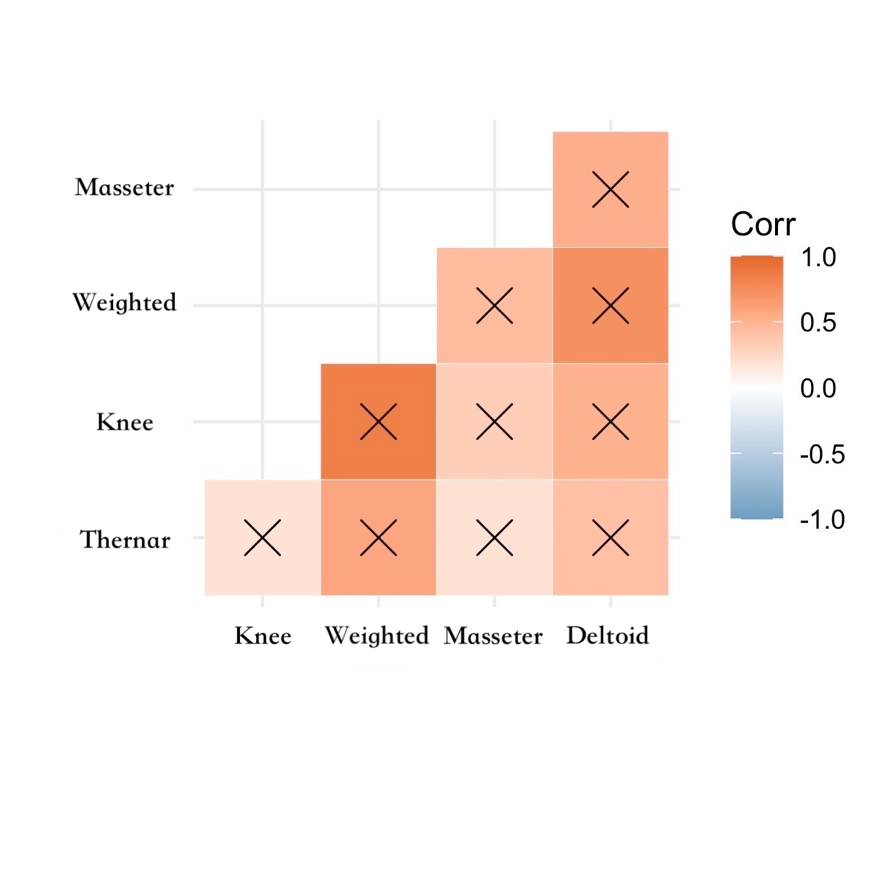


## Fig S3. Correlations between tissue oxygen saturation


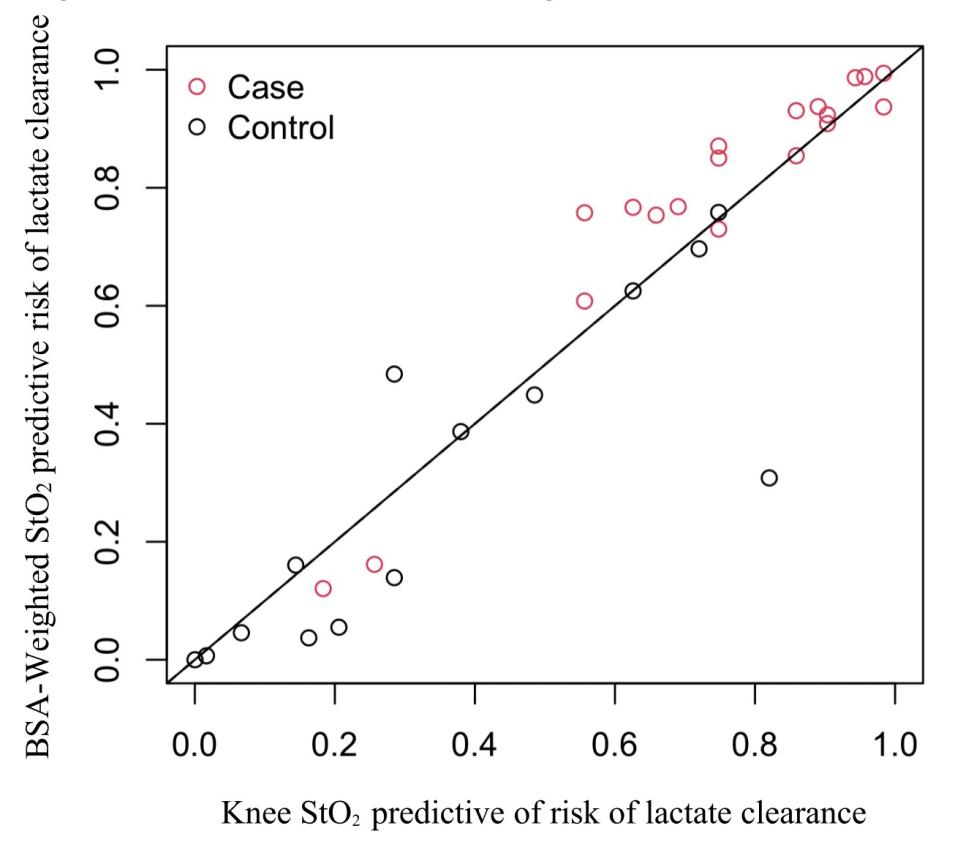


## Fig S4. Predicted probabilities by mean StO_2_ and BSA-weighted StO_2_ with diagonal line showing the comparable predicted probabilities in lactate clearance group and non-clearance group.

The red circles represent the lactate clearance group (case) and the white circles represent the non-clearance group (control). Circles above the diagonal line indicate an increase in the probability of correct prediction of weighted StO_2_ compared to mean StO_2_.


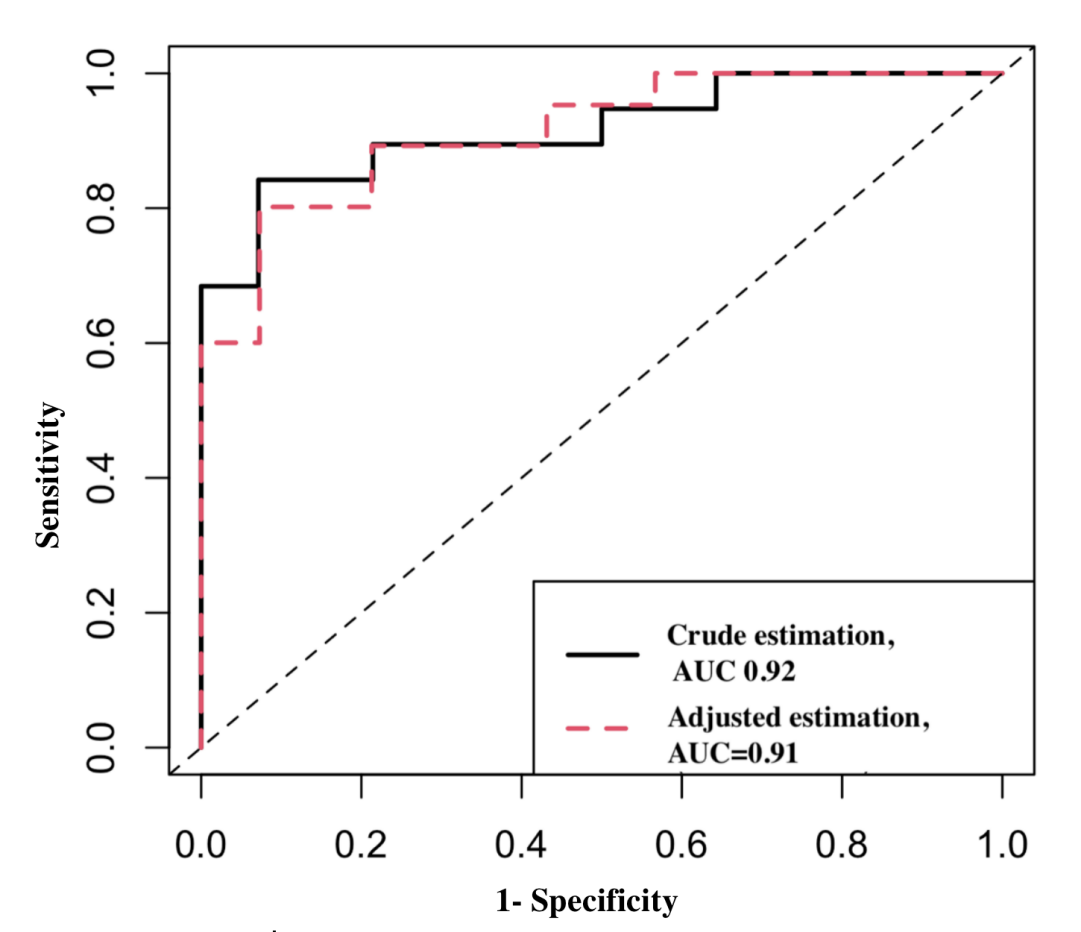


## Fig S5. Adjusted ROC curve.

Black line shows crude receiver operating curve of tissue oxygen saturation predicting 6-hour lactate clearance. Red dashed line shows receiver operating curve adjusted by mean arterial pressure and norepinephrine dose.

# Tables

## Table S1 Comparisons of tissue oxygen saturation between different types of shock

|  | septic shock | cardiogenic shock | hypovolemic shock |
| --- | --- | --- | --- |
| Masseter | 73.8 | 73.7 | 72.5 |
| Deltoid | 75.4 | 74 | 74.5 |
| Thenar | 69.0 | 67.6 | 72 |
| Knee | 70.1 | 65.7 | 73.5 |
| Weighted | 71.7 | 68.7 | 73.3 |

StO_2_ of different sites were presented as mean value.

## Table S2 Correlations between tissue oxygen saturation and MAP, norepinephrine dose

|  | MAP | | norepinephrine dose | |
| --- | --- | --- | --- | --- |
|  | R | P | R | P |
| Masseter | -0.07 | 0.05 | 0.22 | 0.88 |
| Deltoid | -0.31 | <0.01 | 0.06 | 0.73 |
| Thenar | -0.23 | 0.03 | 0.02 | 0.59 |
| Knee | -0.33 | <0.01 | -0.03 | 0.61 |
| Weighted | -0.33 | <0.01 | 0.04 | 0.58 |

## Table S3 Correlations between tissue oxygen saturation and lactate clearance

|  | Patients | R | P |
| --- | --- | --- | --- |
| Masseter | Overall (n = 34) | 0.2 | 0.26 |
|  | Septic shock (n = 24) | 0.069 | 0.75 |
| Deltoid | Overall (n = 34) | 0.42 | 0.013 |
|  | Septic shock (n = 24) | 0.36 | 0.082 |
| Thenar | Overall (n = 34) | 0.21 | 0.24 |
|  | Septic shock (n = 24) | 0.16 | 0.44 |
| Knee | Overall (n = 33) | 0.64 | <0.001 |
|  | Septic shock (n = 23) | 0.53 | 0.010 |
| Weighted | Overall (n = 33) | 0.62 | <0.001 |
|  | Septic shock (n = 23) | 0.49 | 0.018 |
